# Prevalence and predictors of HIV-related stigma among institutional- and community-based caregivers of orphans and vulnerable children living in five less-wealthy countries

**DOI:** 10.1186/1471-2458-10-504

**Published:** 2010-08-19

**Authors:** Lynne C Messer, Brian W Pence, Kathryn Whetten, Rachel Whetten, Nathan Thielman, Karen O'Donnell, Jan Ostermann

**Affiliations:** 1 Center for Health Policy and Inequalities Research; 2812 Erwin Rd, Suite 403; Duke University Campus Box 90392; Durham NC 27705; USA; 2 Center for Health Policy and Inequalities Research; Duke University; Durham NC USA; 3 Duke University; Durham NC USA

## Abstract

**Background:**

In the face of the HIV/AIDS epidemic that has contributed to the dramatic increase in orphans and abandoned children (OAC) worldwide, caregiver attitudes about HIV, and HIV-related stigma, are two attributes that may affect caregiving. Little research has considered the relationship between caregiver attributes and caregiver-reported HIV-related stigma. In light of the paucity of this literature, this paper will describe HIV-related stigma among caregivers of OAC in five less wealthy nations.

**Methods:**

Baseline data were collected between May 2006 through February 2008. The sample included 1,480 community-based and 192 institution-based caregivers. Characteristics of the community-based and institution-based caregivers are described using means and standard deviations for continuous variables or counts and percentages for categorical variables. We fit logistic regression models, both for the full sample and separately for community-based and institution-based caregivers, to explore predictors of acceptance of HIV.

**Results:**

Approximately 80% of both community-based and institution-based caregivers were female; and 84% of institution-based caregivers, compared to 66% of community-based caregivers, said that they would be willing to care for a relative with HIV. Similar proportions were reported when caregivers were asked if they were willing to let their child play with an HIV-infected child. In a multivariable model predicting willingness to care for an HIV-infected relative, adjusted for site fixed effects, being an institution-based caregiver was associated with greater willingness (less stigma) than community-based caregivers. Decreased willingness was reported by older respondents, while willingness increased with greater formal education. In the adjusted models predicting willingness to allow one's child to play with an HIV-infected child, female gender and older age was associated with less willingness. However, willingness was positively associated with years of formal education.

**Conclusions:**

The caregiver-child relationship is central to a child's development. OAC already face stigma as a result of their orphaned or abandoned status; the addition of HIV-related stigma represents a double burden for these children. Further research on the prevalence of HIV-related acceptance and stigma among caregivers and implications of such stigma for child development will be critical as the policy community responds to the global HIV/AIDS orphan crisis.

## Background

The caregiver-child relationship has important long-term consequences for children's health and wellbeing. Attachment theorists consider children to have a need for a secure relationship with an adult caregiver for normal social and emotional development to occur [1,2]. Because orphans and abandoned children (OAC), defined as those who have had one or both parents die or were abandoned by both parents, lack one or more adult caregivers, their healthy development may be compromised.

Caregivers of OAC can contribute to their healthy development. While little research examining the characteristics of OAC caregivers exists, one study considered the reasons caregivers gave for fostering orphans. The authors found that the degree of relatedness to- and financial capacity to care for- an orphan child among the most frequently cited reasons given for caregiving. However, child-level characteristics, like age and gender, were reportedly not considered in the decision to take on the care of an OAC [3]. Most caregivers were found to be female, older, poor, and living without a spouse.

In the face of the HIV/AIDS epidemic that has contributed to the dramatic increase in OAC worldwide [4,5], caregiver attitudes about HIV is one attribute that may affect the quality of OAC caregiving. Caregiver factors that have been previously associated with positive attitudes toward HIV/AIDS, orphans in general, and AIDS orphans in particular include middle age (35-44), polygamy, belief that there are increasing numbers of orphans in the community, having relatives or friends with HIV/AIDS and caregivers' knowledge regarding HIV/AIDS [6]. Concern about becoming infected with HIV/AIDS, however, also negatively affects willingness to serve as a caregiver to AIDS OAC [3].

When the caregiver or community's attitude toward HIV is negative, this attribute may manifest itself in HIV-related stigma. Stigma has been defined as an attribute that is significantly discrediting [7], which can have concrete material consequences. Research indicates stigma alters life chances, including income, employment, housing access, and health [8,9]. Experienced stigma can also result in adverse psychosocial consequences, including distress and depression [10-12]. HIV-related stigma is prevalent. In a recent U.S. study with patients drawn from the HIV Cost and Services Utilization Study, all of the families interviewed recounted experiences with stigma, including 100% of mothers, 88% of fathers, 52% of children, 79% of adult children, and 60% of caregivers. About 97% of families described discrimination fears, 79% of families experienced actual discrimination, and 10% of uninfected family members experienced stigma from association with the parent with HIV [13]. Drivers of stigma include fear, availability and relevance of AIDS-related information, lack of social spaces to engage in dialogue about HIV/AIDS, perceived links between HIV/AIDS, sexual moralities and the control of women and young people, inadequate HIV/AIDS management services, and the ways in which poverty shapes people's reactions to HIV/AIDS [14]. Research suggests that stigma, and the resultant discrimination, can exacerbate the material and psychological problems children already face in the context of the HIV/AIDS pandemic [15-18]. Stigma can prevent proper access to education and care, both directly (through abuse, forced labor and inheritance loss) and indirectly (if children avoid potentially stigmatizing situations such as healthcare and educational interactions) [19]. When stigma is manifested in the OAC-caregiver relationship, through hostility, violence, or differential resource allocation, it can have severe negative impacts on children under care. While very little research has previously considered the extent of stigma expressed against children and youth infected with and affected by HIV/AIDS [20], one author reported significant associations between less expressed stigma behavior and higher knowledge of how to support HIV/AIDS infected persons [21].

In light of the dearth of literature considering caregiver attributes and HIV-related stigma, the goal of this paper is to describe HIV-related stigma among caregivers of OAC in five less wealthy nations.

## Methods

### Study description

The Positive Outcomes for Orphans (POFO) study is a longitudinal cross-cultural research study designed to identify characteristics of care associated with better child outcomes. For the purpose of this work, an orphan or abandoned child (OAC) is defined as a child living without one or more of his/her biological parent. The POFO study is following approximately 2,750 children who were between 6 to 12 years at study outset and their caregivers in 83 institutional care settings and 309 community clusters in six study sites across five less wealthy countries: Cambodia, India (two sites), Kenya, Tanzania, and Ethiopia. Additional study details are located elsewhere [22].

### Study sample

Six geographically defined regions in five counties were chosen to represent cultural, historical, ethnic, religious, political, and geographic diversity. HIV prevalence differed across the countries as well. The 2007 estimated adult (15-49 years of age) HIV prevalence rate for each of the five study countries are as follows: Cambodia (0.8), India (0.3) with Nagaland estimated at more than twice that rate, Tanzania (6.2), Ethiopia (2.1) and Kenya (7.1-8.5) [23]. Two-stage random sampling survey methodology was used to identify a sample of institution- and community-living OAC ages 6 to12 who were statistically representative of the population of institution- and community-living OAC in those regions.

#### Institution Selection

For each of the six study areas, comprehensive lists of all institutions were created. To ensure broad representation, institutions were defined as structures with at least five resident OAC from at least two different families not biologically related to the caregiver(s). The institutional sampling frame was generated through inquiries to local government officials, schools, and organizations working with orphans. Institutions specifically for street children, special needs children, and international adoption were excluded. In total, 83 institutions participated in the study with only three being located in a caregivers home and all having more than five OAC: nine in Battambang, Cambodia (one refused), 13 in Addis Ababa, Ethiopia (two refused), 13 in Kilimanjaro Region, Tanzania (one refused), 14 in Hyderabad, India (five refused), 15 in Dimapur and Kohima Districts of Nagaland, India (two refused), and 21 in Bungoma, Kenya (none refused). Reasons for refusal ranged from fear of psychological damage to the children to wanting monetary compensation for project participation.

#### Selection of Institution-based Children

Institutions provided a list of residential children aged 6 to 12. Using a list of random numbers, up to 20 children per institution were randomly selected. When the target enrollment of 250 children could not be met at a particular site, for instance, in cases when there were not enough institutions from which only 20 children could be selected, then all children in the age range became eligible to participate. Of the 5,243 children cared for by the institutions, 2,396 were reported to be age-eligible, and 1,360 were selected for enrollment. The number of participating children per institution ranged from 1 to 51.

#### Community Sampling Area Selection

The primary community sampling aim was to select an unbiased sample of community-based care settings. In each of the six study areas, 50 sampling areas ("clusters") were selected. Geographic or administrative boundaries were used to define sampling areas, therefore specific definitions varied across sites.

#### Selection of Community-based Children

A community-based OAC was an orphan or an abandoned child (living without either of his/her two parents) not living in an institution. In each sampling area up to five eligible children were selected, either randomly from available lists, or through a house-to-house census conducted until five households with age-eligible children were identified. When the pre-identified sampling areas provided an insufficient number of community-based children, additional sampling areas were substituted; this occurred in 13 villages in Cambodia, 12 in Nagaland, and one in each of the remaining sites. In households with multiple age-eligible children, one child was selected as the child whose first name started with the earliest letter in the alphabet. In total, 1,463 community-based OAC were enrolled in the study.

#### Caregiver Selection

The children's (self-identified) primary caregivers were asked to respond to surveys about themselves and the children. In total, 193 institutional caregivers, ranging from 16 institutional caregivers in Nagaland to 52 in Cambodia, and 1,480 community-based caregivers participated in the assessments.

### Data collection protocol

Baseline data collection was conducted between May 2006 and February 2008 among community-based and institution-based OAC and their caregivers. Four main baseline instruments collected information from: 1) children residing in communities who had a parent who had died or was missing; 2) children residing in institutions; 3) the children's primary caregivers; and 4) a person who could respond to administrative questions about the institution. Age inclusion criteria were based on survey instrument validity and pilot testing. Informed consent was obtained from each participating caregiver and from the heads of participating institutions. Assent was given by all participating children. Interviews were conducted with children and caregivers using their native language in the child's residence and children were interviewed. Ethical approval was provided by the Duke University Institutional Review Board (IRB) and by local and national IRBs in all participating countries.

### Study measures

#### HIV-related stigma and acceptance

Caregivers were asked two questions: "If a relative of yours were sick with HIV, would you be willing to care for him or her"; and "Would you allow your child to play with an HIV-infected child". Caregivers could respond "yes," "no,", or "I don't know."

#### Covariates

Caregivers reported age, gender, marital status, and educational attainment, whether they earned any income (for institution-based caregivers: whether they earned any income outside the institution), and, for institution-based caregivers, the number of years they had been a caregiver. Caregivers also rated their own health on a five-point scale (very good, good, fair, poor, or very poor).

### Analyses

Characteristics of the community-based and institution-based caregivers were described using means and standard deviations for continuous variables or counts and percentages for categorical variables. We fit logistic regression models, both for the full sample and separately for community-based and institution-based caregivers, to explore predictors of acceptance of HIV. The two stigma/acceptance variables were each coded so that the index category (coded as "1") corresponded to a response of "yes" (greater acceptance) while the reference category (coded as "0") corresponded to a response of either "no" or "I don't know." Thus an odds ratio greater than 1 in these models indicates a variable associated with greater acceptance. Logistic models included site fixed effects to account for the six recruitment sites. Analyses were conducted using Stata v.10.1.

## Results

The sample included 1,480 community-based and 192 institution-based caregivers (Table 1). Approximately 80% of both community-based and institution-based caregivers were female, but institution-based caregivers tended to be older (mean age 42 vs. 35 years), more educated (88% with at least some secondary school education compared to 47%), more likely to be never married (43% vs. 6%), more likely to report better health (75% reporting good or very good health vs. 47%) and less likely to be widowed (15% vs. 61%). Eighty-four percent of institution-based caregivers compared to 66% of community-based caregivers said that they would be willing to care for a relative with HIV. A similar disparity was evident for the proportion of caregivers who said they would be willing to let their child play with an HIV-infected child (81% vs. 64%). Only a small proportion of caregivers responded "I don't know" to either question.

**Table 1:** Caregiving-type-stratified characteristics of persons caring for orphans and abandoned children who comprise the POFO cohort

	Mean (SD) or n (%)		
Characteristic	Community caregivers	Institution caregivers	Missing
N	1480	192	
Age, years (range: 11-89)	41.7 (13.5)	35.3 (11.1)	246
Female gender	1147 (84.6%)	146 (77.3%)	158
Years of education			240
0	323 (24.8%)	3 (1.6%)	
1-6	374 (28.7%)	20 (10.8%)	
7-12	552 (42.4%)	101 (54.6%)	
13 or more	53 (4.1%)	61 (33.0%)	
Marital status			20
Never married	85 (5.8%)	80 (42.8%)	
Currently married	388 (26.4%)	63 (33.7%)	
Widowed	898 (61.1%)	28 (15.0%)	
Other	99 (6.7%)	16 (8.6%)	
Number of years as caregiver	N/A	4.4 (7.0)	12
Any earned income*	1039 (71.0%)	53 (30.5%)	40
Caregiver health status			45
Very good	126 (8.7%)	55 (31.1%)	
Good	552 (37.9%)	77 (43.5%)	
Fair	486 (33.4%)	38 (21.5%)	
Poor	258 (17.7%)	7 (4.0%)	
Very poor	35 (2.4%)	0 (0.0%)	
Would care for relative with HIV			80
Yes	934 (65.5%)	142 (83.5%)	
No	369 (25.9%)	21 (12.4%)	
Don't know	123 (8.6%)	7 (4.1%)	
Would allow child to play with HIV+ child			76
Yes	921 (64.4%)	137 (81.1%)	
No	408 (25.5%)	26 (15.4%)	
Don't know	101 (7.1%)	6 (3.6)	

* For institutional caregivers: Any earned income outside institution

In a multivariable model predicting willingness to care for an HIV-infected relative, adjusted for site fixed effects (Table 2), being an institution-based caregiver appeared associated with greater willingness than community-based caregivers. Decreased willingness was reported by older respondents (adjusted odds ratio (AOR) and 95% Confidence Interval (95% CI) = 0.9 [0.8, 1.0] for each 10-year age increment) and those reporting no education (AOR = 0.4; 95% CI: 0.2, 0.6) while willingness increased with high levels of formal education (13 plus years). There was some suggestion that willingness increased with better health (AOR = 1.2; 95% CI: 1.0, 1.4) and varied by marital status, with currently married respondents being the most willing to care for an HIV-infected relative. In models stratified by caregiver setting (institution vs. community-based), older age was predictive of decreased willingness among community-based but not among institution-based caregivers. Low education (none) was associated with reduced willingness among both community- and institution-based caregivers, while better health was associated with greater acceptance among institution-based caregivers only. In most models currently married caregivers reported greater willingness than others, although the precision of these estimates was reduced in the stratified-model setting.

**Table 2:** Combined and caregiving-type-stratified multivariable predictors of stigma among POFO caregivers: willingness to care for an HIV-infected relative.

	Would care for relative with HIV		
	All caregivers	Community-based	Institution-based
Institution-based caregiver	1.74 (0.94, 3.20)	N/A	N/A
Age, per 10 years	0.87 (0.76, 1.00)	0.82 (0.71, 0.95)	1.23 (0.81, 1.87)
Female gender	0.77 (0.51, 1.18)	0.75 (0.48, 1.18)	1.08 (0.29, 3.97)
Years of education			
None	0.37 (0.24, 0.56)	0.41 (0.26, 0.63)	0.05 (0.00, 0.99)
1-6	0.81 (0.54, 1.21)	0.85 (0.56, 1.30)	1.10 (0.15, 8.24)
7-12	1.00 (ref)	1.00 (ref)	1.00 (ref)
13 or more	2.02 (1.08, 3.78)	2.41 (1.08, 5.38)	2.33 (0.68, 7.93)
Marital status			
Currently married (ref)	1.00 (ref)	1.00 (ref)	1.00 (ref)
Never married	0.63 (0.34, 1.14)	0.45 (0.22, 0.93)	1.42 (0.41, 4.87)
Widowed	0.64 (0.44, 0.92)	0.58 (0.39, 0.86)	0.58 (0.12, 2.85)
Other	0.42 (0.22, 0.81)	0.39 (0.19, 0.80)	0.69 (0.11, 4.35)
Health (on 5-point scale)	1.21 (1.01, 1.44)	1.14 (0.94, 1.37)	2.55 (1.20, 5.43)
Any earned income	0.83 (0.57, 1.21)	0.74 (0.50, 1.11)	2.26 (0.46, 11.17)
Years as caregiver (#)	N/A	N/A	0.29 (0.03, 2.73)

In the multivariable, site-fixed-effects models predicting willingness to allow one's child to play with an HIV-infected child (Table 3), female gender and older age was associated with less willingness (AOR = 0.8; 95% CI: 0.7, 0.9 and AOR = 0.6; 95% CI: 0.4, 1.0, respectively). Willingness was also positively associated with years of formal education, with high education (13 plus years) being associated with increased odds of willingness. In the models stratified by caregiving location, young age and six or fewer years of education was associated with decreased willingness among community-based, but not among institution-based caregivers. Thirteen or more years of education was associated with increased willingness in both community-based and institution-based settings (AOR = 3.4; 95% CI: 1.5, 7.6 and 3.4; 95% CI: 1.1, 11.0, respectively).

**Table 3:** Combined and caregiving-type-stratified multivariable predictors of stigma among POFO caregivers: willingness to let child play with an HIV-infected child.

	Would allow child to play with HIV+ child		
	All caregivers	Community-based	Institution-based
Institution-based caregiver	0.77 (0.42, 1.40)	N/A	N/A
Age, per 10 years	0.82 (0.71, 0.93)	0.78 (0.68, 0.90)	0.95 (0.64, 1.41)
Female gender	0.63 (0.41, 0.97)	0.63 (0.40, 1.01)	0.71 (0.20, 2.52)
Years of education			
None	0.26 (0.17, 0.39)	0.27 (0.18, 0.42)	0.37 (0.02, 6.00)
1-6	0.53 (0.35, 0.80)	0.51 (0.33, 0.79)	1.13 (0.21, 6.17)
7-12	1.00 (ref)	1.00 (ref)	1.00 (ref)
13 or more	3.70 (1.95, 7.03)	3.40 (1.52, 7.59)	3.43 (1.06, 11.02)
Marital status			
Currently married (ref)	1.00 (ref)	1.00 (ref)	1.00 (ref)
Never married	0.56 (0.31, 1.02)	0.51 (0.24, 1.09)	0.66 (0.21, 2.05)
Widowed	0.84 (0.58, 1.20)	0.79 (0.54, 1.16)	0.60 (0.14, 2.68)
Other	0.62 (0.32, 1.20)	0.62 (0.29, 1.29)	0.45 (0.09, 2.33)
Health (on 5-point scale)	1.05 (0.88, 1.26)	1.03 (0.85, 1.24)	1.41 (0.78, 2.58)
Any earned income	1.21 (0.82, 1.79)	1.02 (0.67, 1.55)	4.51 (0.96, 21.18)
Years as caregiver (#)	N/A	N/A	0.39 (0.07, 2.27)

## Discussion

In this paper, we considered two indicators of HIV-related stigma in a sample of nearly 2,000 caregivers of orphans and abandoned children (OAC) in five less wealthy nations: willingness to care for a relative with HIV/AIDS in one's home and willingness to let one's child play with a child who is HIV-infected. Overall, relatively small proportions of caregivers reported HIV-related stigma, with less stigma reported by institution-based caregivers compared to community-based caregivers. More years of formal education was associated with greater caregiver acceptance (less stigma) among all caregivers. Older age and female gender were associated with decreased acceptance among community-based but not institution-based caregivers.

The dramatic increase in the number of OAC worldwide, particularly as a result of the AIDS epidemic, has focused attention on the importance of the caregiving setting and the caregiver-OAC relationship in influencing child outcomes. HIV-related stigma is widespread and could plausibly affect the caregiver-OAC relationship for children infected with or orphaned by HIV. Yet no other literature exploring attributes of OAC caregivers and the relation of those attributes to HIV-related stigma was identified. Recent reviews by UNICEF, Save the Children (UK) and other agencies have underscored this critical gap in the literature. "In spite of ample anecdotal and descriptive evidence that HIV/AIDS-related stigma and discrimination are affecting children, not enough systematic research has been done to illustrate the nature and extent of the problem, and how it relates to other key sources of disadvantage for children in poor, high-prevalence areas" [24]. Recent policy statements have recommended de-emphasizing institution-based care in favor of community-based care settings on the basis that better child care will result [25,26]; in the present study, however, we found greater acceptance and less reported stigma among institution-based compared to community-based caregivers. If HIV-related stigma affects OAC caregiving, this finding may be important to the ongoing policy discussion.

Reducing stigma and quality HIV care are related. Preliminary data from research in rural Haiti suggest that the introduction of quality HIV care can lead to a rapid reduction in stigma, with resulting increased uptake of testing [27]. Other research suggests that people who live in a context where they see people with HIV/AIDS being treated with kindness and care are far more likely to acknowledge their vulnerability, to seek out information about how to protect themselves or go for voluntary counseling and testing, and to take precautions in their sexual relationships [14]. Therefore, it appears that stigma reduction and HIV care work synergistically to improve prevention and reduce risk.

Our ability to describe the prevalence and predictors of two markers of HIV stigma in a large sample of OAC caregivers from five less wealthy nations is a strength of this study. However, this work is also not without limitations. While caregivers were asked about HIV-related stigma, it is not clear if the OAC they were caring for were HIV-infected or if their parents had died from AIDS or AIDS-related complications. Prior qualitative work has indicated that in many parts of the world, AIDS-related deaths are often not reported as such by family members or friends, who instead may cite other causes of death, such as malaria or diarrhea [14]. We also were not able to examine whether caregivers' reports of HIV-related stigma was associated with differential child outcomes over time. Longitudinal data would shed important light on this question.

## Conclusion

The caregiver-child relationship is central to a child's development. OAC already face stigma as a result of their orphaned or abandoned status; OAC affected by HIV, either by virtue of being infected themselves or because they were orphaned due to HIV, face a potential double burden of stigmatization. And many, if not most OAC, are not even aware of their HIV status or the status of their parents. Therefore, caring for OAC often carries with it the uncertainty of the child's status.

Stigma and stigmatization have been linked to reinforcement of social inequalities of class, race, gender, and sexuality [28]. While most caregivers reported willingness, or the absence of stigma, over 25% of caregivers willingly reported on their HIV-related stigma. Further research on the prevalence of HIV-related acceptance and stigma among caregivers, and the implications of such stigma for healthy child development, will be critical as the policy community develops appropriate responses to the global AIDS orphan crisis.

## List of abbreviations

HIV: human immunodeficiency virus; AIDS: acquired immunodeficiency syndrome; OAC: orphaned and abandoned child/children; AOR: adjusted odds ratio; 95% CI: 95 percent confidence interval; UNICEF: United Nations Children's Fund; UK: United Kingdom.

## Competing interests

The authors declare that they have no competing interests.

## Authors' contributions

LCM: conceived of the analysis and wrote the manuscript. BWP: co-conceived of the analysis, conducted the statistical analysis and helped write the manuscript. KW: conceived of the study and helped to draft the manuscript. RW: conceived of the study, participated in the study design and coordination. NT: participated in the study design and coordination and helped to draft the manuscript. KOD: participated in the study design and coordination and helped to draft the manuscript. JO: participated in the study design and coordination and prepared data for analysis. All authors read and approved the final manuscript

## Pre-publication history

The pre-publication history for this paper can be accessed here:

http://www.biomedcentral.com/1471-2458/10/504/prepub
